# Betahistine for residual dizziness after canalith repositioning in benign paroxysmal positional vertigo: a systematic review of randomized controlled trials

**DOI:** 10.3389/fphar.2026.1855843

**Published:** 2026-06-02

**Authors:** Esteban Ortiz-Prado, Jorge Vasconez-Gonzalez, Sandra Gavilanes-Rodriguez, Melissa Castillo-Bustamante, Valeria Barreto, Mónica Maya-Castro, Juan S. Izquierdo-Condoy

**Affiliations:** 1 One Health Research Group, Universidad de Las Americas, Quito, Ecuador; 2 Ear and Vertigo Clinic, Quito, Ecuador; 3 Universidad Pontificia Bolivariana, Vertigo and Dizziness Center in Medellín, Medellín, Colombia; 4 Postgraduate Program in Otolaryngology, Universidad Central Del Ecuador, Quito, Ecuador; 5 Department of Otolaryngology, Faculty of Medicine, Universidad de Las Americas, Quito, Ecuador

**Keywords:** benign paroxysmal positional vertigo, betahistine, canalith repositioning maneuvers, randomized controlled trials, residual dizziness

## Abstract

**Background:**

Benign paroxysmal positional vertigo (BPPV) is the most common peripheral vestibular disorder. Although canalith repositioning maneuvers (CRMs) are highly effective, a substantial proportion of patients experience residual dizziness (RD) after successful repositioning. Betahistine has been proposed as an adjunctive therapy to facilitate recovery; however, its effectiveness in RD remains uncertain.

**Aims:**

To evaluate the efficacy and safety of betahistine in adults with BPPV who experience residual dizziness after canalith repositioning maneuvers.

**Methods:**

A systematic review was conducted in accordance with the PRISMA 2020 guidelines. PubMed/MEDLINE, Scopus, and Web of Science were searched from inception to 1 February 2026. Randomized controlled trials including adults with BPPV who were treated with CRMs and presented with residual dizziness were eligible. Betahistine administered as adjunctive therapy was compared with placebo, no treatment, maneuver alone, or other pharmacological interventions. Two reviewers independently performed study selection, data extraction, and risk of bias assessment using the JBI checklist. Due to heterogeneity, findings were synthesized narratively.

**Results:**

Four randomized controlled trials published between 2012 and 2022, including approximately 418 participants, were included. Betahistine daily doses ranged from 36 to 48 mg/day, with treatment durations of 1–4 weeks. Outcomes assessed included symptom intensity (VAS), disability (DHI), participation measures, duration of RD, and clinical resolution. Two trials reported significant improvements in symptom reduction, disability scores, or probability of RD resolution with betahistine, whereas two studies found no clear differences in primary outcomes or final clinical resolution compared with controls. Methodological quality varied, with one study considered at low risk of bias and three presenting uncertainties in key domains.

**Conclusion:**

Betahistine may provide symptomatic or functional improvement in selected patients with residual dizziness after CRMs, but current randomized evidence remains limited, heterogeneous, and methodologically uncertain. Routine or universal use is not supported. Further adequately powered trials with standardized RD definitions, comparable outcomes, longer follow-up, and objective vestibular assessments are needed to clarify its clinical role.

**Systematic Review Registration:**

https://www.crd.york.ac.uk/PROSPERO/view/CRD420261339853.

## 1 Introduction

Benign paroxysmal positional vertigo (BPPV) is the most common peripheral vestibular disorder, accounting for approximately 20% of all cases of vertigo worldwide (von Brevern et al., 2007). The term was introduced by Dix and Hallpike to describe a benign condition characterized by brief, paroxysmal episodes of vertigo triggered by changes in head position (Koshi and Sutton, 2025). BPPV primarily affects adults, with a peak incidence between the fifth and sixth decades of life, and an estimated annual incidence ranging from 10.7 to 64 cases per 100,000 inhabitants (Kim and Zee, 2014; M et al., 2007).

BPPV results from displacement of otoconia—calcium carbonate crystals—from the utricle into the semicircular canals. These particles may remain free (canalithiasis) or adhere to the cupula (cupulolithiasis), disrupting endolymph flow and causing abnormal vestibular stimulation (Koshi and Sutton, 2025). The posterior canal is involved in about 60%–90% of cases due to its anatomical relationship with gravity and the utricle. Although most cases are idiopathic (M et al., 2007; Power et al., 2020), BPPV may also occur secondary to head trauma, vestibular neuritis, or other inner ear disorders (Riga et al., 2011).

Canalith repositioning maneuvers (CRMs) constitute the first-line treatment and are supported by high-level evidence (White et al., 2005). Among these, the Epley maneuver achieves symptom resolution in more than 90% of cases by facilitating the return of otoconia to the utricle, where they can be reabsorbed or eliminated (Gupta and Solanki, 2022). Despite its high efficacy, a considerable proportion of patients (31%–61%) experience persistent symptoms after successful repositioning. Although BPPV may resolve spontaneously within a few months, up to half of patients require active treatment and may experience a prolonged recovery (Özgirgin et al., 2024; Seok et al., 2008).

A clinically relevant and increasingly recognized condition is residual dizziness (RD), defined as persistent nonspecific dizziness, instability, or lightheadedness after resolution of positional vertigo and in the absence of typical positional nystagmus following CRMs (Kingma et al., 2026). Reported prevalence ranges from 23% to 70%, and RD is associated with reduced quality of life, increased fall risk, and delayed functional recovery (Ke et al., 2022). Its mechanisms are likely multifactorial, including persistent utricular dysfunction, residual otoconial debris insufficient to elicit nystagmus, sensory mismatch after abnormal canal stimulation, delayed central adaptation, and age-related degenerative changes (Kingma et al., 2026). In BPPV, central vestibular compensation should not be understood in the same way as compensation after fixed unilateral vestibular hypofunction. Rather, it may reflect central recalibration after transient abnormal canal stimulation, sensory mismatch, and persistent subclinical vestibular input after apparent mechanical resolution of positional vertigo. Residual utricular dysfunction, small amounts of otoconial debris insufficient to provoke positional nystagmus, and maladaptive postural or perceptual responses may all contribute to persistent dizziness despite a negative positional test. This process may be influenced by patient mobilization, vestibular rehabilitation, emotional state, and the capacity of the central nervous system to adapt to a new functional status (Kingma et al., 2026; Özgirgin et al., 2024).

Another challenge in studying residual dizziness after BPPV treatment is the lack of a universally accepted operational definition. In the literature, RD has been described using heterogeneous criteria, which complicates comparison across studies. Recently, Kingma et al. proposed a refined framework for BPPV-related residual dizziness and introduced the CLEAR algorithm to support its recognition. In this review, we focused on patients with persistent dizziness after successful repositioning maneuvers, consistent with the classical form of BPPV-related RD (Kingma et al., 2026).

**FIGURE 1:**
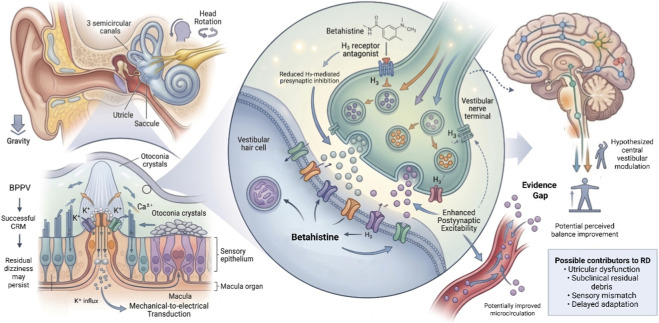
Proposed mechanisms and translational evidence gap for betahistine in post-maneuver recovery after BPPV. The diagram summarizes anatomical and cellular pathways involved in vestibular signaling and illustrates the hypothesized actions of betahistine, including H_3_ receptor antagonism, reduced H_3_-mediated presynaptic inhibition, potential modulation of vestibular signaling, and potential improvement of inner-ear microcirculation. These mechanisms provide a biological rationale for its potential use in residual dizziness after canalith repositioning maneuvers; however, they were not directly validated by the randomized trials included in this review, which mainly assessed clinical or patient-reported outcomes rather than objective measures of vestibular compensation.

While CRMs correct the mechanical component of BPPV, they do not directly address residual symptoms potentially related to sensory mismatch, utricular dysfunction, or delayed central adaptation. Therefore, pharmacological strategies aimed at improving post-maneuver recovery have been proposed. Betahistine, a histamine analog acting as a weak H1 agonist and H3 antagonist, is thought to improve inner-ear microcirculation and modulate vestibular nuclei activity; however, its role in facilitating central compensation in BPPV-related RD remains mechanistically plausible but clinically unconfirmed (Della Pepa et al., 2006) (Figure 1).

Clinical studies suggest that betahistine may reduce symptom burden and improve quality of life in some peripheral vestibular disorders, with a favorable safety profile (Hui et al., 2022; Parfenov et al., 2017).

However, despite its widespread use, evidence on the effectiveness of betahistine for residual dizziness in BPPV remains limited and heterogeneous (Alsolamy et al., 2025; Li et al., 2023). Studies vary in design, patient selection, outcomes, and follow-up, and no consensus exists regarding its role as adjunctive therapy after CRMs. Given the frequency and clinical impact of residual symptoms, a systematic synthesis of the evidence is warranted.

This systematic review aimed to evaluate the efficacy and safety of betahistine in adults with BPPV who experience residual dizziness after canalith repositioning maneuvers, compared with control or alternative treatments, focusing on short-term symptomatic relief, functional improvement, and safety.

## 2 Materials and methods

### 2.1 Design and reporting

A systematic review was conducted in accordance with the PRISMA 2020 recommendations (Page et al., 2021). The review protocol was registered in PROSPERO under the registration number CRD420261339853.

### 2.2 Information sources and search strategy

The search was performed in PubMed/MEDLINE, Scopus, and Web of Science (WoS) from database inception to 1 February 2026. A systematic literature search was conducted using both controlled vocabulary and free-text terms related to the clinical condition, the intervention, and the outcome of interest.

The strategy combined, using Boolean operators, terms related to the condition of interest (“benign paroxysmal positional vertigo” or “BPPV”), the intervention (“betahistine” or “betahistina”), repositioning maneuvers (“canalith repositioning,” “repositioning maneuver/manoeuvre,” “Epley,” “Semont,” “Lempert”), and the clinical outcome of interest (“residual dizziness” or “persistent dizziness”). The search was restricted to the presence of these terms in titles and abstracts. Filters were applied for human studies and randomized controlled trials (RCTs), with no date restrictions. Additionally, reference lists of included studies were manually screened to identify further relevant literature.

### 2.3 Eligibility criteria

Eligibility criteria were defined according to the PICO framework.

Population (P): Adults (≥18 years) diagnosed with BPPV, treated with canalith repositioning maneuvers, and presenting residual dizziness at follow-up.

Operational definition of residual dizziness: Persistence of nonspecific dizziness (e.g., instability, floating sensation, lightheadedness, or imbalance) after resolution of typical positional vertigo following the maneuver.

Intervention (I): Betahistine administered as adjunctive therapy during or after repositioning maneuvers.

Comparator (C): Placebo, no treatment, maneuver alone, or other pharmacological treatments.

Study design (S): Randomized controlled trials (RCTs) in humans.

Reviews, editorials, letters, conference abstracts, and case reports were excluded, as were studies that did not assess residual dizziness as an outcome; duplicate publications; studies without full-text access or insufficient information to confirm eligibility; and studies in which the effect could not be attributed to betahistine due to non-comparable cointerventions between groups.

### 2.4 Study selection process

Study selection was conducted in two stages. First, two reviewers independently screened titles and abstracts to identify potentially eligible studies. Second, full texts of selected records were reviewed to confirm final eligibility. Discrepancies were resolved by consensus or through consultation with a third reviewer.

**FIGURE 2:**
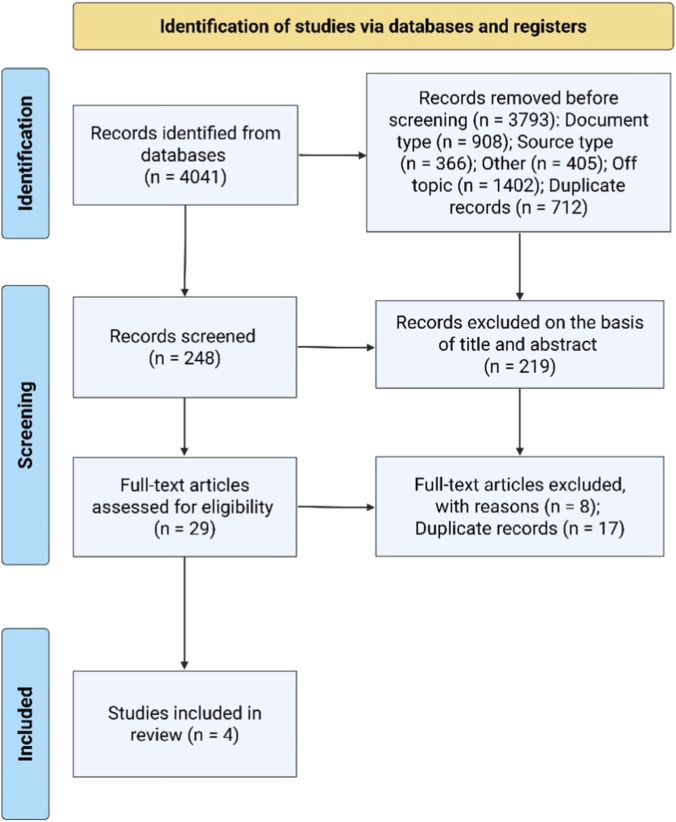
PRISMA flow diagram of manuscript selection for this systematic review.

A total of 4,041 records were identified through database searching. Of these, 3,793 records were removed prior to screening (document type, source type, other reasons, off-topic, and duplicates), leaving 248 records for title and abstract screening. After this phase, 219 records were excluded, and 29 full-text articles were assessed for eligibility. Subsequently, 17 duplicate reports were identified and removed by comparing titles, authors, DOI numbers, publication years, and journal information across databases. Eight additional articles did not meet the inclusion criteria, resulting in four randomized controlled trials included in the qualitative synthesis (Figure 2).

### 2.5 Data extraction

Two reviewers independently extracted data using a standardized and previously piloted form. Information collected included author and year, country, sample size, baseline participant characteristics, diagnostic criteria for BPPV, type and number of maneuvers performed, betahistine regimen (dose, frequency, and duration), comparator characteristics, instruments used to quantify symptoms and disability (e.g., DHI and/or VAS), follow-up duration, reported outcomes, and adverse events. Discrepancies were resolved by consensus and, when necessary, by consultation with a third reviewer.

### 2.6 Risk of bias assessment

Methodological quality of the included trials was assessed using the JBI Critical Appraisal Checklist for Randomized Controlled Trials (Barker et al., 2023). Two reviewers independently evaluated key domains, including randomization method, allocation concealment, baseline comparability, blinding (participants, personnel, and outcome assessors where applicable), handling of attrition, consistency in outcome measurement, and appropriateness of statistical analysis. Disagreements were resolved by consensus.

### 2.7 Evidence synthesis

Given the limited number of included studies and variability in comparators, outcome measures, and follow-up time points, findings were integrated through a structured narrative synthesis. The direction and magnitude of effect were described according to reported clinical outcomes (symptom relief, functional improvement, and safety), highlighting consistency across studies and the main sources of clinical and methodological heterogeneity.

## 3 Results

Following the selection process illustrated in Figure 2, 29 studies were evaluated. Of these, 17 were excluded as duplicates and eight for not meeting eligibility criteria. Ultimately, four studies—all randomized controlled trials—were included in the qualitative synthesis of this review (Guneri and Kustutan, 2012; Jalali et al., 2020; Sayin et al., 2022; Wu et al., 2021). The four included studies were published between 2012 and 2022 and comprised approximately 418 adult participants diagnosed with BPPV and treated with repositioning maneuvers. Sample sizes ranged from 72 to 129 patients, with mean ages around the fifth decade of life.

In all studies, betahistine was administered as adjunctive therapy following repositioning maneuvers, with total daily doses ranging from 36 to 48 mg/day and treatment durations ranging from 1 to 4 weeks. Comparators included maneuver alone, placebo, or no treatment.

Evaluated outcomes included symptom intensity (VAS), functional disability (DHI), quality of life or activity participation, duration of residual dizziness, and clinical resolution.

### 3.1 Effects of betahistine on symptoms and disability

Findings across the included studies were heterogeneous. Two trials—Sayin et al. in 108 patients with posterior canal BPPV and Jalali et al. (2020) in 117 patients with residual dizziness after successful repositioning—reported significant benefits of betahistine in terms of symptom reduction, functional improvement, or higher likelihood of residual dizziness resolution (Table 1).

**TABLE 1:** Characteristics of the included studies in the systematic review.

Study (year)	Country/Center	Design	Population (age) and BPPV	RD definition	Betahistine intervention	Comparator	N (randomized/analyzed)	Follow-up	Main reported outcomes	Key findings
Wu et al. (2021)	China, single center	RCT, 3 arms	129 patients with BPPV and RD after successful CRP	RD after successful CRP	Betahistine 12 mg orally TID for 4 weeks	(1) Vestibular rehabilitation 4 weeks; (2) control without specific treatment	129/129 (43 per group)	4 weeks + symptom duration analysis	Primary: VAP (activities/participation). Secondary: SOT (balance), RD duration	Betahistine did not improve VAP vs. control (p = 0.28). No differences in SOT. Median RD duration 14 days (betahistine) vs. 19 days (control), NS (p = 0.40)
Sayin et al. (2022)	Turkey	RCT, 2 arms	Posterior canal BPPV confirmed by dix–Hallpike and upbeat torsional nystagmus; ≥18 years; excludes other vestibular disorders and major comorbidities	RD not explicitly defined; assessed symptomatic/disability improvement post-maneuver	Epley + betahistine 24 mg orally BID (48 mg/day); duration until 1-week control	Epley alone	108 randomized/100 completed (8 lost)	1 week; second maneuver if dix–Hallpike positive	VAS and DHI pre- and at 1 week; nystagmus resolution (dix–Hallpike)	Overall epley success 95% (96% vs. 94%). VAS and DHI improved in both groups; greater reduction with betahistine: Final VAS 0.74 vs. 1.92; final DHI 3.16 vs. 9.88; significant differences (p < 0.001)
Jalali et al. (2020)	Iran, 2 hospitals (guilan univ.)	Double-blind RCT, 3 arms	117 patients aged 18–65 years, posterior canal BPPV (canalithiasis) confirmed; only those with positive RD after successful CRP included	RD: Lightheadedness/dizziness/unsteadiness without vertigo or nystagmus after CRP resolution	Betahistine (16mg, three times/day; 1-week intervention)	Placebo and dimenhydrinate arm	117/117	1 week	RD symptoms (by components), DHI (categories), mBBS, logistic regression for “no RD”	Significant model (p < 0.04): Betahistine associated with higher probability of “no RD” vs. placebo (OR 3.18). No adverse events reported
Guneri and Kustutan (2012)	Turkey (ministry of health approval)	Double-blind RCT, 3 arms (placebo-controlled)	72 patients aged 18–79 years, unilateral posterior canal BPPV (canalithiasis) confirmed; no vestibular suppressants/ototoxic drugs; no CNS pathology/prior surgery	Assessed post-treatment symptoms and QOL	Epley + betahistine 24 mg orally BID (48 mg/day) for 1 week	Epley alone; epley + placebo BID 1 week	72/72	1 week (and reassessment at 2 weeks if persistent)	DHI, VADL, european evaluation of vertigo, vertigo symptom scale; persistence with positive dix–Hallpike	Post-treatment final scores similar between groups, but greater percentage symptom reduction with betahistine. Persistence at 1 week: 10/72 (13.8%), no differences; all resolved after additional week

In contrast, Wu et al. (2021), in 129 patients with residual dizziness after successful maneuvers assigned to vestibular rehabilitation, betahistine (12 mg three times daily), or no treatment for 4 weeks, found no differences between betahistine and control (p = 0.28). Median residual dizziness duration was 14 days in the betahistine group and 19 days in the control group, with no significant differences (p = 0.40). Finally, the trial by Guneri et al. in 72 patients with posterior canal BPPV observed a greater percentage reduction in symptoms with betahistine but no differences in final outcomes or clinical resolution (Table 1).

Variations in dose, treatment duration, definitions of residual dizziness, and outcome measures limited comparability across studies and supported the decision to conduct a qualitative synthesis rather than a meta-analysis.

### 3.2 Risk of bias assessment

Risk of bias was assessed using the JBI tool for randomized controlled trials (Table 2). Overall, the available evidence derives from trials of variable methodological quality, with one study considered at low risk of bias. Jalali et al. (2020) demonstrated the most robust profile, with consistent fulfillment of key domains (randomization, allocation concealment, baseline comparability, participant blinding, and intention-to-treat analysis), suggesting a low overall risk of bias. The remaining three studies presented important uncertainties in critical domains such as sequence generation, allocation concealment, blinding, and intention-to-treat analysis (Table 2). Therefore, although outcomes were generally measured consistently and statistical analyses were mostly appropriate, the overall certainty of the evidence should be interpreted with caution, and the review conclusions should emphasize the need for more rigorous and better-reported trials.

**TABLE 2:** JBI critical appraisal checklist for randomized controlled trials.

Authors	Year	Q1	Q2	Q3	Q4	Q5	Q6	Q7	Q8	Q9	Q10	Q11	Q12	Q13
Wu et al.	2021	Unclear	Unclear	Unclear	Yes	Yes	Unclear	Yes	Unclear	Unclear	Yes	Yes	Yes	Unclear
Sayin et al.	2022	Unclear	Unclear	Unclear	No	Unclear	Unclear	Yes	Yes	Unclear	Yes	Yes	Yes	Yes
Jalali et al.	2020	Yes	Yes	Yes	Yes	Unclear	Unclear	Yes	Yes	Yes	Yes	Yes	Yes	Yes
Guneri & kustutan	2012	Yes	Yes	Unclear	Yes	Yes	Unclear	Yes	Unclear	Unclear	Yes	Yes	Yes	Unclear

Questions of JBI Critical Appraisal Checklist for Randomized Controlled Trials. Q1 True randomization used? Q2 Allocation concealment? Q3 Groups similar at baseline? Q4 Participants blinded? Q5 Those delivering treatment blinded? Q6 Outcome assessors blinded? Q7 Groups treated identically other than intervention? Q8 Follow-up complete/differences described and analyzed? Q9 Intention-to-treat analysis? Q10 Outcomes measured same way for groups? Q11 Outcomes measured reliably? Q12 Appropriate statistical analysis used? Q13 Trial design appropriate/deviations accounted for?

## 4 Discussion

In this systematic review, we sought to clarify the efficacy and safety of betahistine in adults with BPPV who present residual dizziness after canalith repositioning maneuvers. Our search identified only four randomized controlled trials evaluating betahistine as adjunctive therapy following repositioning maneuvers in adults with persistent symptoms compatible with residual dizziness. Overall, the results suggest a possible clinical benefit signal that is highly dependent on study design, the definition of residual dizziness, the instrument used for its measurement, and likely the duration of treatment. Two trials (Jalali et al., 2020; Sayin et al., 2022) reported significant improvements in patient-centered outcomes, such as symptom intensity (VAS), disability (DHI), or the probability of “no residual dizziness.” In contrast, Wu et al. (2021) found no differences compared with control for the primary outcome (VAP), and Guneri and Kustutan (2012) observed mainly a greater percentage reduction in symptoms, without clear differences in final outcomes or clinical resolution between groups. This heterogeneous pattern, together with the limited number of available studies, small sample sizes, and methodological limitations of the included trials, suggests that the current evidence on the utility of betahistine for residual dizziness after repositioning maneuvers remains insufficient to draw firm conclusions or to establish definitive clinical recommendations.

An important translational limitation is that the mechanistic rationale for betahistine should not be interpreted as being directly validated by the available trials. Although betahistine is biologically plausible as a facilitator of vestibular compensation through H_3_ antagonism, modulation of vestibular nuclei activity, and improved inner-ear microcirculation, the included RCTs mainly assessed clinical or patient-reported outcomes, including VAS, DHI, participation measures, duration of RD, and clinical resolution. None of the studies systematically measured central vestibular compensation using dedicated vestibular function tests such as vHIT, caloric testing, vestibular evoked myogenic potentials, or longitudinal objective vestibular biomarkers. Therefore, the current evidence supports only a possible symptomatic benefit in selected patients, not confirmation of the proposed compensatory mechanism. Future trials should integrate objective vestibular testing with standardized RD outcomes to bridge this mechanistic-clinical gap.

The findings of our qualitative synthesis are consistent with previous literature. However, our review differs from previous meta-analyses because it applied a narrower PICO focused specifically on randomized trials evaluating betahistine in the context of residual dizziness after canalith repositioning, rather than broader BPPV populations treated with Epley maneuver plus betahistine. This distinction is important because improvement in acute BPPV symptoms or DHI scores after repositioning does not necessarily indicate resolution of true RD as a distinct post-maneuver syndrome. For example, meta-analyses focused on posterior canal BPPV have suggested that the combination of maneuver plus betahistine may improve disability scores (e.g., DHI) in some settings, whereas other analyses have not demonstrated consistent differences in resolution or overall “efficacy,” particularly when follow-up is short (Alsolamy et al., 2025; Li et al., 2023). In the study conducted by Alsolamy et al., three studies were also identified in which no significant differences were observed when betahistine was added to the treatment (Acar et al., 2015; İnan and Kıraç, 2019; Ugurlu et al., 2012). One possible explanation for these discrepancies is that the potential effect of betahistine may be time-dependent: trials assessing very early outcomes (≈1 week) may underestimate benefits that could become more evident when the drug is maintained for several weeks, especially if its primary mechanism is to facilitate central vestibular compensation or alleviate nonspecific symptoms rather than to modify the mechanical resolution of BPPV. In our analysis, treatment duration ranged from 1 to 4 weeks, with total daily doses between 36 and 48 mg/day, and this variability may explain part of the inconsistency across results. Additionally, it is important to highlight the heterogeneity in the definition of residual dizziness. According to Kingma et al., two types of residual dizziness (RD) have been described. Type 1 BPPV-related RD is defined as an initial presentation with typical BPPV nystagmus that resolves after one or more canalith repositioning maneuvers (CRM), with residual dizziness symptoms reported at the follow-up appointment. In contrast, Type 2 RD refers to patients who present dizziness symptoms without positional nystagmus, but with a clear history of BPPV that was not examined or diagnosed during the acute phase and that apparently resolved spontaneously (Kingma et al., 2026). Based on this, it can be observed that Jalali et al. included patients whose BPPV was confirmed using either the supine head-roll test or the Dix–Hallpike maneuver. Sayin et al. included patients presenting with upbeat torsional nystagmus elicited by the Dix–Hallpike maneuver. Wu et al. included BPPV patients with residual dizziness following successful canalith repositioning procedures (CRP), while Guneri et al. included patients with posterior semicircular canal (PSC) BPPV of the canalithiasis type diagnosed using the Dix–Hallpike maneuver. This lack of standardization in diagnostic criteria and definitions of residual dizziness may also have influenced the outcomes reported across the studies (Guneri and Kustutan, 2012; Jalali et al., 2020; Sayin et al., 2022; Wu et al., 2021).

Using the Kingma et al. framework (Kingma et al., 2026), Jalali et al. and Wu et al. most closely approximate Type 1 BPPV-related RD, because patients had confirmed BPPV, underwent successful repositioning, and subsequently reported residual non-vertiginous dizziness. In contrast, Sayin et al. and Guneri & Kustutan did not operationally define RD as a distinct post-repositioning syndrome and primarily assessed post-treatment symptom or disability improvement. Therefore, these studies should be interpreted as indirect evidence rather than fully comparable RD populations.

Residual dizziness after a successful maneuver is common and may persist as instability or lightheadedness even in the absence of positional vertigo or nystagmus. Its origin is multifactorial, combining peripheral mechanisms (utricular dysfunction, residual debris, microcirculatory factors) and central processes (delayed compensation/adaptation), in addition to comorbidities such as anxiety and cardiometabolic factors that may amplify symptoms (Hui et al., 2022). In this context, betahistine may act as a symptomatic adjunct and, potentially, as a modulator of vestibular recovery, but its effect is expected to be modest and variable if residual dizziness is not a homogeneous entity but rather a clinical scenario encompassing a wide range of symptoms and underlying mechanisms across patients.

Our work also highlights a central methodological issue: the lack of standardization in the operational definition of residual dizziness after repositioning maneuvers and in its measurement. This conceptual heterogeneity may also explain the limited number of randomized trials identified in our review, as many studies evaluating interventions in BPPV do not clearly distinguish between persistent vertigo due to incomplete repositioning and nonspecific dizziness after successful maneuvers. While some trials focused on general disability scales (DHI) or symptom intensity (VAS), Wu et al. prioritized participation and activities (VAP) and incorporated objective balance measures (SOT). This heterogeneity in outcome assessment, together with differences in populations (exclusion criteria, BPPV subtypes, comorbidities), comparators (placebo, maneuver alone, no treatment), and very short evaluation periods in some cases, limits direct comparability of findings and justifies the methodological decision to perform a qualitative synthesis rather than a meta-analysis. Additionally, risk of bias assessment revealed uncertainties in critical domains—including randomization and allocation concealment, blinding, and intention-to-treat analysis—in three of the four trials (Guneri and Kustutan, 2012; Sayin et al., 2022; Wu et al., 2021), requiring cautious interpretation of reported effects, particularly given moderate sample sizes and short follow-up.

From a clinical perspective, the available evidence suggests that betahistine may be considered, in selected scenarios, as an adjunct to alleviate symptoms or reduce perceived disability after repositioning maneuvers in BPPV, but not as an intervention with uniform efficacy (Kingma et al., 2026). In practice, the decision to use it should be individualized, taking into account symptom severity, functional impact, tolerance, comorbidities, and, importantly, the need to exclude alternative diagnoses (vestibular migraine, Ménière disease, central or cardiovascular causes) and to integrate non-pharmacological strategies such as vestibular rehabilitation, which in some studies appears to have a clearer effect on participation and functional performance (Alolayet and Murdin, 2025).

Finally, our findings underscore the need for future research in patients with residual dizziness after successful canalith repositioning maneuvers. Multicenter trials with standardized definitions of RD, clinically meaningful and comparable outcomes, longer follow-up, transparent methodological reporting according to CONSORT, and stratification by relevant risk factors, including age, anxiety, cardiometabolic comorbidities, vitamin D status, and osteopenia, are required. Future studies should also incorporate objective vestibular assessments, such as vHIT, caloric testing, VEMPs, posturography, or other measures of vestibular compensation, to determine whether betahistine has a true mechanistic effect beyond symptomatic improvement. Only with better-designed and adequately reported studies will it be possible to define more precisely the role of betahistine, including optimal dose and duration, in the management of residual dizziness associated with BPPV.

## 5 Conclusion

Current randomized evidence suggests that betahistine may provide symptomatic or functional benefits in selected adults with residual dizziness following canalith repositioning maneuvers for BPPV; however, the available data remain insufficient to support its universal recommendation as an adjuvant treatment. Any use should be individualized, considering symptom severity, functional impact, tolerance, comorbidities, and exclusion of alternative diagnoses. Heterogeneous results, methodological limitations, variability in RD definitions and outcome measures, differences in treatment duration, and overall study quality preclude definitive conclusions. The findings of this systematic review should therefore be considered exploratory and hypothesis-generating. Well-designed trials with adequate sample sizes, standardized RD criteria, longer follow-up, and objective vestibular assessments are needed to clarify its precise clinical role.

## Data Availability

The original contributions presented in the study are included in the article/supplementary material, further inquiries can be directed to the corresponding author.
